# Online Testing Yields the Same Results as Lab Testing: A Validation Study With the False Belief Task

**DOI:** 10.3389/fpsyg.2021.703238

**Published:** 2021-10-13

**Authors:** Lydia Paulin Schidelko, Britta Schünemann, Hannes Rakoczy, Marina Proft

**Affiliations:** Department of Developmental Psychology, University of Göttingen, Göttingen, Germany

**Keywords:** online studies, validation study, developmental psychology, psychology methods, Theory of Mind, false belief

## Abstract

Recently, online testing has become an increasingly important instrument in developmental research, in particular since the COVID-19 pandemic made in-lab testing impossible. However, online testing comes with two substantial challenges. First, it is unclear how valid results of online studies really are. Second, implementing online studies can be costly and/or require profound coding skills. This article addresses the validity of an online testing approach that is low-cost and easy to implement: The experimenter shares test materials such as videos or presentations via video chat and interactively moderates the test session. To validate this approach, we compared children’s performance on a well-established task, the change-of-location false belief task, in an in-lab and online test setting. In two studies, 3- and 4-year-old received online implementations of the false belief version (Study 1) and the false and true belief version of the task (Study 2). Children’s performance in these online studies was compared to data of matching tasks collected in the context of in-lab studies. Results revealed that the typical developmental pattern of performance in these tasks found in in-lab studies could be replicated with the novel online test procedure. These results suggest that the proposed method, which is both low-cost and easy to implement, provides a valid alternative to classical in-person test settings.

## Introduction

Developmental research largely depends on collecting data from children. While varying in methods, set-ups and concrete testing sites, so far, most research has been conducted in an interpersonal, face-to-face setting between an experimenter and a child. Thus, with the beginning of the COVID-19 pandemic, most well-established testing routines were suddenly disrupted and the need for new, safe, and contact-free ways to test children for developmental studies arose.

In the last decade, online testing for psychological research already became more and more prominent for adult studies, with several thousand participants taking part in social science experiments every day on platforms like *Amazon Mechanical Turk* (*MTurk*) and *Prolific* (Bohannon, 2016). More recently, developmental researchers have started to establish first online platforms for children, including *Lookit* (Scott and Schulz, 2017; Scott et al., 2017) and *Discoveries Online* (Rhodes et al., 2020), that both use an unmoderated set-up (where children and families do not interact with the researchers), and *TheChildLab.com* (Sheskin and Keil, 2018) that uses a moderated set-up (where the experimenter calls the families via video chat). However, existing platforms and paradigms are not always available for everyone, because of high costs (e.g., for the experimental testing software), programming requirements (e.g., JavaScript), mandatory software downloads or data protection regulations of the software that do not align with the policies of the research institution. Against this background, when we had to close our lab in March 2020, we decided to establish our own moderated testing paradigm for children. In this article, we want to present this novel set-up and validate it as a suitable, safe and broadly accessible tool for online data collection with children.

In our paradigm, we video call families via the software *BigBlueButton* (BBB) and the experimenter then interacts with the children with the help of animated videos or slides. The combination of BBB and screen-sharing comes with several advantages. Concerning the software, BBB is a free, open source, on-premises software. Additionally, once it is established, it comes with low technical requirements both on side of the experimenter as well as the participant as it runs in all common browsers. Furthermore, the servers for BBB are hosted locally, in our case in our institute. Thus, the use of this software allows researchers to adhere to the highest data protection standards, since only the host can access usage and meta-data. Note, however, that while using BBB offers clear advantages, our general set-up is not limited to BBB but is in principle applicable to almost every video chat software that allows screen sharing.

Having set up a technically suitable paradigm, the most pressing question concerns the data quality that can be obtained by testing children with it. Is our moderated online paradigm really appropriate for (remote) data collection? To answer these questions, we wanted to validate our method. Specifically, we tested whether we can conceptually replicate the effects found in in-lab face-to-face settings in analogous studies implemented in our new online paradigm. Importantly, to avoid population-based effects that could explain potential differences between online and in-lab testing, we drew the samples for both paradigms from the same population: our database of parents who had previously given consent to participate with their children in developmental studies. Both samples were thus comparable concerning (a) socio-demographic variables (age and gender were measured, but the sample is also likely to be comparable concerning other socio-demographic variables, e.g., living environment, as the database only includes families living in and around the same city), (b) familiarization with developmental studies (86% of the children participating in an online study participated in at least one other in-person study in our lab before), and (c) incentive structure (we did not directly compensate parents or children for either paradigm).

For the comparison of the two methods, we used a well-established social-cognitive task: the standard false belief (FB) task (Wimmer and Perner, 1983). The FB task is designed to tap children’s ability to attribute subjective mental states to others and is generally seen as the litmus test for having a Theory of Mind (ToM). In its standard version, children see a vignette (acted out with puppets) in which an agent puts an object in one of two boxes and leaves the scene. In her absence the object is transferred to the other box and children are then asked to predict where the agent will look for her object upon her return. Results from countless live studies show that children typically start to master this verbal version of the FB task around the age of four, with younger children falsely predicting that the agent will look for her object where it really is (see Wellman et al., 2001). In addition, we administered the structurally analogous true belief (TB) version of the task. Originally designed to control for extraneous task demands in the FB version, recent studies reported a paradoxical picture: once children master the FB task, they begin to fail the TB task. The TB and FB tasks are thus highly negatively correlated between 3 and 5 such that children first pass the TB and fail the FB task and then show the reverse pattern (see Fabricius et al., 2010; Perner et al., 2015; Oktay-Gür and Rakoczy, 2017). This strange effect in the TB task does not seem to document a conceptual limitation, though. One possibility is that it rather reflects children’s sensitivity to task pragmatics that they develop on the basis of their growing Theory of Mind. Several studies reveal that the more advanced in ToM children are, the more pragmatically sensitive they become, and the more they get confused by the triviality of the TB test question given the shared perspective of the experimenter and the child (“Why is the experimenter asking me such as stupid question? I guess there must be a more complex answer than the obvious one”; see Oktay-Gür and Rakoczy, 2017; Rakoczy and Oktay-Gür, 2020). In line with the idea of a high pragmatic component of the TB effect, once the task is modified to become less pragmatically confusing (either by converting it into non-verbal format, or by changing the context so that the question now is less trivial) the effect goes away and children perform competently from age 3 onward without any decline in performance. This is highly relevant for present purposes as it shows that the TB test question in its standard version seem to present a very sensitive measure of children’ susceptibility to task pragmatics. The TB task therefore lends itself perfectly as a very stringent test for the comparability of live vs. online testing in even subtle respects of verbal interaction and interpretation.

To validate our online set-up, we thus compare children’s performance in the two testing formats (in-lab and online): Do the two paradigms lead to comparable results? This question is not trivial. In fact, the existing literature suggest that there are several indicators that (moderated) online testing might indeed lead to different results. On a general level, there is the video deficit effect (VDE): the phenomenon that children solve the same task later and less accurately when the task is presented in a video than when it is presented by a person (Anderson and Pempek, 2005). The VDE has been found for a variety of tasks such as word-learning (e.g., Thierry and Spence, 2004), object-retrieval (e.g., Troseth and DeLoache, 1998) and imitation (e.g., Klein et al., 2006). Recently, it has also been documented for the FB task: 4- and 5-year-old (who usually pass the task) failed to correctly predict the agent’s behavior when the story was presented on a video (Reiß et al., 2014, 2019). Furthermore, there is first data from TheChildLab.com concerning moderated online testing more specifically (Sheskin and Keil, 2018). While in general children tested online provided expected answers on a variety of classical tasks, the FB task seemed to be especially hard: Only 9- to 10-year-olds reliably solved the task, while the 5- to 8-year-olds performed at chance level, opening a gap of around 4–5 years compared to standard in-lab testing results.

For the present validation project, we thus collected data on 3- to 4-year-old children’s FB and TB understanding in two online studies and compared it to data we obtained from previous in-lab studies with closely matched protocols. Data from in-lab testing was collected pre-COVID and (partly) reanalyzed for the purpose of the current study (for more details, see Supplementary Material). In Study 1, we compared children’s performance on the standard FB task between the in-lab and online test setting. In Study 2, we widened the focus and tested whether children’s more complex performance patterns in TB and FB tasks would differ between in-lab and online test setting.

## Study 1

### Methods

#### Participants

The final sample includes 112 monolingual German speaking children aged 36–58 months (mean age = 44.28 months; 56 girls; 64 of them tested in an online test session [mean age = 44.22 months; 31 girls (48%)]; 48 [mean age = 44.35 months; 25 girls (52%)]^1^ in an in-lab test session). Mean age did not differ between settings [*t*(110) = 0.120, *p* = 0.905]. All children live in and around the same medium sized German university town, that is generally characterized by mixed socio-economic backgrounds^2^. Six additional children were tested but not included in data analyses because of uncooperative behavior (online setting: *n* = 3), technical issues during the test session (online setting: *n* = 1), parental interference during the test session (online setting: *n* = 1) and language issues^3^ (in-lab setting: *n* = 1). Children in this and the subsequent study were recruited from a databank of children whose parents had previously given consent to experimental participation.

#### Design

All children received two trials of a standard change-of-location FB task. The order and direction of location change (from left to right or vice versa) of the trials were counterbalanced. The tasks were presented either as videos in an online testing format or acted-out in an in-lab setting (for comparable scripts and stimuli and a detailed overview of how the online and in-lab tasks were implemented, see Supplementary Material).

#### Materials and Procedure

##### False belief task

In the FB task (Wimmer and Perner, 1983), Protagonist A (for example, the boy) placed his object (for example, his ball) in one of two boxes (box 1). In his absence, protagonist B (for example, the girl) moved the ball to the other box (box 2) and the experimenter (E) asked the test question “When the boy returns, where will he look for the ball first?” (Correct answer: box 1) (For additional control questions, see Supplementary Material).

#### Set-Up

##### Moderated online study

In the online test setting, one female experimenter (E) presented the tasks remotely (on a computer screen, no smartphone) via a video conferencing platform (mainly BigBlueButton, in case of technical issues: *Zoom*). During the test session, the child and E communicated via audio and video streaming. The story lines of the FB task were visually implemented as short video clips (created with the animation software *VyondTM* © *2021 GoAnimate*). The child watched the video clips while E told the story lines. At the end of each story line, E asked the control and test questions.

##### In-lab study

In the in-lab setting, children were tested in single sessions by two female experimenters in the laboratory. E1 first acted out the FB task with little figures and then asked the control and test questions.

### Results and Discussion

**FIGURE 1:**
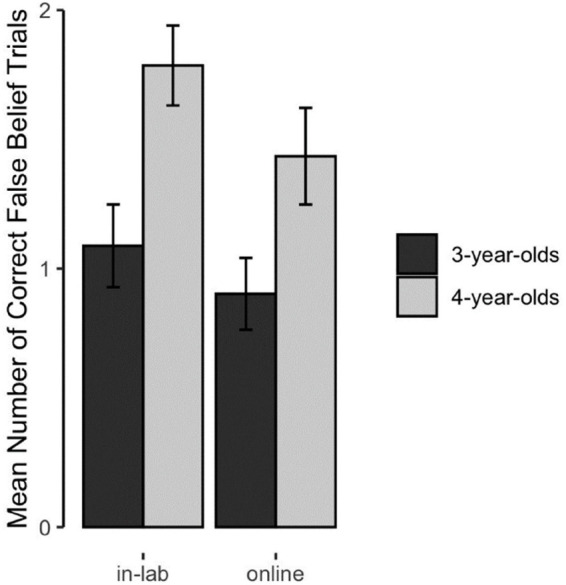
Mean number of correctly answered false belief trials. This figure depicts the mean number of correctly answered FB trials (0–2) as a function of test setting and age (error bars depict ±1 SE).

Figure 1 shows children’s performance on the FB test question as a function of age and test setting. In accordance with the literature, we would expect that children’s performance on the standard FB task increases with age. If the test setting has an impact on children’s performance in this task, there should be a difference between settings most likely in that the effect of age on children’s performance should be different between settings.

For this reason, we set up a Generalized Linear Mixed Model with binomial error structure and a logit link function. As dependent variable, we included children’s success on each test trial. To test for an effect of setting and whether the effect of age on performance is different between settings, we included test setting and age measured in months^4^ and their interaction. To account for repeated measures, we included children’s ID as random intercept effect. We checked for the model’s stability by calculating estimates after case wise exclusion of participants. This revealed a stable model. We also checked for multicollinearity (all *VIF*s ≤ 1.001).

We compared this full model to a null model which included age and the random intercept. This comparison was not significant (likelihood ratio test: χ^2^ = 0.509, *df* = 2, *p* = 0.775). Likewise, a closer look at the model revealed that the interaction effect of test setting and age was not significant (*b* = −0.840, *p* = 0.567). Also, the main effect for test setting was not significant (*b* = −0.297, *p* = 0.813). Only the main effect for age was significant (*b* = 3.789, *p* = 0.013). Thus, in accordance with the literature, children’s performance increased with age. However, in which setting, in-lab or online, the study was conducted did not impact children’s performance.

## Study 2

### Methods

#### Participants

Seventy-six 36- to 53-month-old native German speaking children were included in the final sample (mean age = 43.76 months; 38 girls). Forty-nine children were tested in an online test setting [mean age = 43.49 months; 23 girls (46%)]. Twenty-seven [mean age = 44.26 months, 15 girls (56%)]^5^ were tested in an in-lab test setting. Mean age did not differ between settings [*t*(74) = 0.605, *p* = 0.547]. The children live in and around the same medium sized German university town, that is generally characterized by mixed socio-economic backgrounds^6^. Five additional children were tested in the online test setting but excluded from analysis because they were uncooperative (*n* = 4) or had severe language issues (e.g., could not follow the story line and the experimenter’s questions; *n* = 1).

#### Design

Children again received two trials of a standard change-of-location FB task. Additionally, they received two trials of the TB condition. The two trials of a condition (FB or TB) were presented in blocks. The order of the two blocks and sides of the two trials within the blocks were counterbalanced. The tasks were presented either as an animated slide show in an online testing format or acted-out in an in-lab setting (for comparable scripts and a detailed overview of how the online and in-lab tasks were implemented, see Supplementary Material).

#### Material and Procedure

##### False belief and true belief task

The protocol was slightly adapted from the classic change-of-location task by Wimmer and Perner (1983) used in Study 1 in that E placed the object in the box and moved the object from the first to the second location in the protagonist’s absence (FB) or after her return (TB). After that (TB) or after the protagonist’s return (FB), E asked the test question “Where does the protagonist think that the toy car is?” [Correct answer: box 1 (FB), box 2 (TB)] (For additional control questions, see Supplementary Material).

#### Set-Up

##### Moderated online study

The same set-up was used as in Study 1. The tasks were presented in a slide show, which was displayed on the child’s screen via the platform’s screen sharing function. While the child was watching the animated slide show, E told the child the story line and asked the control and test questions.

##### In-lab study

In the in-lab format, children were tested as in Study 1 in single sessions by one of five experimenters in the laboratory or in a quiet room of children’s day care.

### Results and Discussion

**FIGURE 2:**
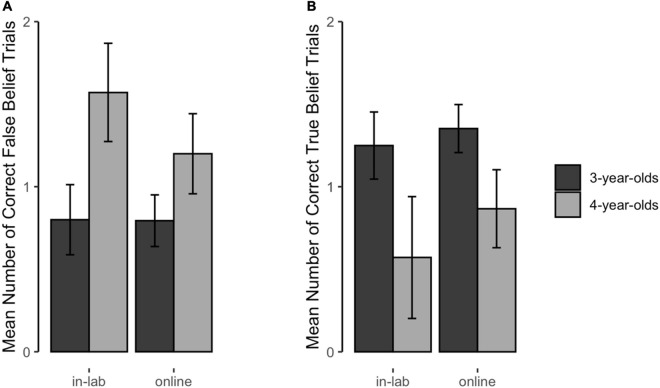
Mean number of correctly answered false **(A)** and true **(B)** belief trials. This figure depicts the mean number of correctly answered FB **(A)** and TB **(B)** trials (0–2) as a function of test setting and age (error bars depict ±1 SE).

Figure 2 shows children’s performance on the FB (a) and TB (b) test questions as a function of age and test setting. In accordance with the literature, we would expect an interaction between age and the belief type: Children performance on the FB task increases with age while it decreases for the TB task. If the test setting has an impact, this interaction of age and belief type should be different between settings.

Again, we set up a Generalized Linear Mixed Model with binomial error structure and a logit link function and success on test trial as dependent variable. To test for the effect of test setting on the interaction of age and belief type, we included test setting, age and belief type and their interactions in the model. To account for repeated measures, we included children’s ID as random intercept effect. The model was stable and not multicollinear (all *VIF*s = 1). Again, we compared this full model to a null model. The null model included age, belief type, their interaction and the random intercept.

This full-null model comparison was not significant (likelihood ratio test: χ^2^ = 2.312, *df* = 4, *p* = 0.679). Likewise, a closer look at the model revealed neither a significant 3-way-interaction of test setting, age and belief type (*b* = 0.616, *p* = 0.268), nor any interaction with test setting (with age: *b* = −0.196, *p* = 0.606; with belief type: *b* = 0.453, *p* = 0.376). Also, there was no main effect for test setting (*b* = −0.177, *p* = 0.617). In contrast, the interaction effect of age and belief type was significant (*b* = −1.656, *p* < 0.001). Thus, in accordance with the literature, children’s performance increased with age for the FB task and decreased for the TB task. The test setting did not have an impact.

## General Discussion

Here, we present and validate a new moderated online testing paradigm for developmental studies. In this paradigm we call families via the video chat software BigBlueButton where the experimenter then interacts with the child with the help of animated videos or slides. The main question regarding the validity of this paradigm was whether it yields results comparable to and converging with in-lab methods. To address this question, we directly compared children’s performance in this online paradigm with data from pre-COVID in-lab testing in a standard false belief (FB) and matching true belief (TB) task (Wimmer and Perner, 1983; Oktay-Gür and Rakoczy, 2017). Importantly, we drew samples for both methods from the same database. Thus, all participants were drawn from one population and live in the same local environment. Moreover, in-lab and online samples were matched for age and gender. This reduced potential population-based effects and allowed us to compare the two methods in a very direct and stringent way.

We found no differences between the two testing formats. First, in both studies, 3- and 4-year-old’s performance in the online FB task was equivalent to their performance in the acted-out in-lab versions of the task as well as to what we would expect in that age range given the widely documented “4-year-revolution” of mastering standard FB tasks (Perner, 1991; Wellman et al., 2001). Second, in accordance with previous studies, we found a characteristic performance pattern in FB and TB tasks such that children with age become more proficient in the former while becoming less proficient in the latter. This pattern held equally in both testing formats, with no difference between the in-lab and online tests.

By using our moderated online testing paradigm, we thus replicated children’s performance from in-lab testing in samples that were drawn from the same population and without facing issues of data loss. Crucially, however, our paradigm does not only seem to closely match interpersonal, face-to-face testing in terms of “cold” indicators such as data quality. Moreover, it also seems to resemble live set-ups in terms of the naturalness and pragmatics of the interaction: when asked a trivial test question (about an agent’s true belief), children showed the same response patterns in the online and the live version. One possible interpretation based on recent research (Rakoczy and Oktay-Gür, 2020) is that children were equally prone to draw pragmatic inferences based on their shared perspective with the experimenter, and fall prey to pragmatic confusions in the online setting as in the in-lab setting. In conclusion, our method seems to be a valid and promising instrument for developmental research.

At the same time, the present results leave open many crucial questions. First, in contrast to previous work (e.g., Anderson and Pempek, 2005; Reiß et al., 2014), we found no indication of the video deficit effect (VDE). Thus, watching video presentations (as children did in our Study 1) does not always seem to disrupt children’s performance in comparison to live demonstrations. But why didn’t the well-documented VDE occur in our paradigm? What is the crucial difference between the cases in which a VDE occurs (in many previous studies) and cases in which it does not (like the present one)? So far, we can only speculate. One crucial difference between the online format and “classical” video presentations is that in our online paradigm the video and the experimenter are both on the screen while in the classic version only the video is presented on-screen with the experimenter sitting next to the child as a live interaction partner who asks test questions. Thus, while in the classic format the child has to handle two parallel worlds (on-screen and live), in the online version all relevant information is presented on-screen, potentially helping the child to encode the video more easily. Other potential influencing factors might be related to the sample (including children’s age and their drastically increased familiarity with media use during the pandemic) or the specific task type (see Strouse and Samson, 2021). More future research is needed to systematically test the different conditions under which the VDE occurs in relation to online research.

Second, again in contrast to previous work (Sheskin and Keil, 2018), we found no difference in children’s relative performance on belief tasks between online and in-lab settings. Thus, administering the task in a moderated testing paradigm *per se* does not seem to negatively influence children’s performance. But then, why were there these gaps in previous work? What is the difference between those cases in which online testing is detrimental to performance and those, like the present one, in which it is not? Again, so far, we can only speculate. When we compare our studies to previous ones, at least two differences emerge: Sheskin and Keil (2018) only presented color coded pictures to the children, whereas we implemented a step-by-step analogous video (or animated slide show) version of the acted-out task version using carefully designed online stimuli [e.g., an animated human hand acting out the change of location and (pre-recorded) verbal interaction between protagonists in the story line onscreen and the experimenter; for more details on scripts and stimuli, see Supplementary Material]. This suggests that subtle details of online implementations might matter. Another crucial difference is that we had the opportunity to directly compare the data we obtained from the two methods (online and live) rather than loosely contrasting online data to effects from the literature. For this direct comparison we drew the samples from the same population, while previous studies mostly document a more diverse, broader distributed sample in their online compared to in-lab studies (see also Rhodes et al., 2020). Given these differences, it seems plausible to assume that previous work might have underestimated children’s performance in (moderated) online paradigms due to population-based effects. Note, however, that although our samples were drawn from the same population, we cannot exclude selective processes in our studies either. There might be a some sort of selection regarding which parents of our population agreed to online testing. Such processes might have led to a less diverse sample and an overestimation of children’s performance. Future research is needed to address this possibility and systematically test for the effects of different population-specific parameters such as socio-economic status, living environment, mobility, closeness to the research institute or time flexibility. For example, even though samples for online and in-lab studies are drawn from the same general population (database), do the sub-samples that respond to live vs. online study invitations differ in subtle demographic respects? Also, note that absence of evidence for a difference between in-lab and online testing of belief tasks, of course, does not amount to evidence of absence of any such potential differences. Future research needs to investigate more systematically and stringently whether there is really no effect of test setting. Such an approach of Bayesian null hypothesis testing will require a larger sample than the current one and will be possible once children can be tested again in an in-lab setting.

The overarching aim of the present project was to find a method for testing children online that is secure, low-cost and easy to implement while yielding comparable results to interpersonal, face-to-face in-lab testing. The results of our two validation studies suggest that with our moderated online testing paradigm we successfully designed such a tool. Future work should now focus on developing the tool further, especially testing its suitability concerning different task types (e.g., more interactive ones that require spontaneous interventions by the child) or different dependent variables (e.g., pointing or eye-tracking). Hopefully, the broader implementation and development of this paradigm then paves the way for more online research in the future, as it has the potential to make developmental research more accessible to a wider audience of participants and researchers.

## Data Availability Statement

The raw data supporting the conclusions of this article are available in the Supplementary Material.

## Ethics Statement

Ethical review and approval was not required for the study on human participants in accordance with the Local Legislation and Institutional Requirements. Written informed consent for participation was not provided by the participants’ legal guardians/next of kin because parts of the studies were conducted online. In the online studies, parents/legal guardians gave verbal consent before the testing was started. Verbal consent was recorded and stored separately from the recording of the test session. For the studies conducted in the laboratory or day care, parents/legal guardians gave written consent.

## Author Contributions

MP, LS, BS, and HR contributed to conception and design of the study. LS did part of the data collection. BS performed the statistical analysis. MP, LS, and BS wrote the sections and first draft of the manuscript. HR supervised the planning and execution process, provided resources for the data collection, and gave critical review and commentary on the draft of the manuscript. All authors contributed to manuscript revision, read, and approved the submitted version.

## Conflict of Interest

The authors declare that the research was conducted in the absence of any commercial or financial relationships that could be construed as a potential conflict of interest.

## Publisher’s Note

All claims expressed in this article are solely those of the authors and do not necessarily represent those of their affiliated organizations, or those of the publisher, the editors and the reviewers. Any product that may be evaluated in this article, or claim that may be made by its manufacturer, is not guaranteed or endorsed by the publisher.

## Footnotes

1 The original sample from Schünemann et al. (2022) included sixty-one 2 1/2–4 1/2-year-old children. For the purpose of the current study, we reduced the data set to a relevant subset of children between 3 and 4 1/2 years (for more details, see Supplementary Material).

2 Note that we did not collect any data on race, educational level or socio-economic background.

3 For the original purpose of the lab study, children were required to be monolingual German (see Schünemann et al., 2022).

4 Age was z-standardized.

5 The original sample from Oktay-Gür and Rakoczy (2017, *Exp. 2*) included 171 participants. For the purpose of the current study, we reduced the data set to the relevant subset (for more details, see Supplementary Material).

6 Note that we did not collect any data on race, educational level, or socio-economic background.
