# Supplementary material for: Online Testing Yields the Same Results as Lab Testing: A Validation Study With the False Belief Task
Source: Front Psychol. 2021 Oct 13;12:703238. doi: 10.3389/fpsyg.2021.703238 (PMC8548716; doi:10.3389/fpsyg.2021.703238)
Supplement: Supplementary file 1 [file Table_1.pdf]

Supplementary Online Material (SOM)

Methods

Detailed descriptions of subsamples

The present data consists of two sets of studies (Study 1 and Study 2) each consisting of one pre-Covid in-lab study and an online study with matching protocols. The studies also include published data that were (partly) reanalyzed for the purpose of the current study.

The data from online testing in Study 1 were collected as part of a project investigating children's understanding for different subjective mental states that is not published yet. The data were reanalyzed for the purposes of this study. The original sample from this study consisted of sixty-four 36- to 53-month-old children that were all included in the analysis of the current study.

The data from in-lab testing in Study 1 were collected before the Covid-19 pandemic as part of another project investigating children's understanding for different subjective mental states (Schünemann, Schidelko et al., 2022) and partly reanalyzed for the purposes of this study. The original sample from Schünemann, Schidelko and colleagues (2022) included sixty-one 30- to 58-month-old children. For the new research question and in order to match the mean age of the sample tested in the online setting with the sample tested in the lab test setting, we reduced the sample of children tested in lab to 36- to 58-month-old children (*M*(*online*)= 44.22 months, *M*(*in lab*)= 44.31 months). For the original purpose of the lab study, children were required to be monolingual German (*see* Schünemann, Schidelko et al., 2022).

The data from online testing in Study 2 were collected for the purpose of the current study. The sample included children between 36 and 53 months so that it allowed the inclusion of children who have and have not developed an explicit false belief understanding, yet (Wellman et al., 2001).
The data from in-lab testing in Study 2 were collected before the Covid-19 pandemic as part of a project investigating children's understanding for true and false beliefs (Oktay-Gür & Rakoczy, 2017, Exp. 2) and partly reanalyzed for the purposes of this study. The original sample from Oktay-Gür and Rakoczy (2017, *Exp. 2*) included 171 participants (children between 37 and 143 months and adults). First, we reduced the data set to the relevant subset of participants who were tested in the (here relevant) standard version of the task. Second, to match the mean age of the sample tested in the online-setting with the sample tested in the lab test setting, we needed to reduce the sample tested in lab to 37- to 53-month-old children (*M*(*online*) = 43.48 months, *M*(*in lab*) = 44.26 months). Additional to the original sample of 171 participants tested in the lab, 14 children were tested but excluded from data analysis because they were uncooperative (*n* = 2), due to insufficient linguistic abilities (*n* = 2), or due to experimental error (*n* = 10). Unfortunately, we can no longer trace which of these children belonged to the age group and test condition relevant here.

Table 1

Detailed procedure of study 1

		Study 1	
		In Lab	Online
Script	Introduction	• E1: Now, I show you a story. Look, here are two boxes. And this is the boy *(E1 shows little figurine).*<br>• E1 acts Boy: Hello!<br>• E1: The boy is playing with his ball *(E1 acts Boy playing with the ball).*<br>• E1*:* But now, he is bored of playing all alone. The boy puts the ball in the blue box and leaves for the playground *(E1 acts Boy putting the ball in blue box and leaving the scene).*	• E1: I'll show you a story now and I'll tell you what is happening in the story *(E1 starts playing the silent video).*<br>• E1: Look, there is a house and two boxes. One blue box and one purple box.<br>• E1: And now, Tine comes with her ball *(Girl with ball in her hands enters the scene).*<br>• E1: Tine is thirsty now, so she wants to go inside to drink something. She puts her ball into the blue box. Then, she goes into the house.
	Location change	• E1: The boy is on the playground now. He is far away and cannot see and cannot hear us anymore.<br>• E1: Now, the girl comes *(E1 acts second figurine coming into the scene).* The girl takes the ball from the blue box and puts it into the green box *(E1 acts girl moving ball from blue to green box).*<br>• E1: Then, the girl leaves again *(E1 acts Girl leaving the scene)*	• E1: Tine is in the house now to drink something. She cannot see what is happening here.<br>• E1: Then, a boy, Felix, comes *(Boy comes from the left/right side into the scene).* Felix takes the ball out of the blue box and puts it into the red box. Then, Felix leaves *(Boy puts ball from blue into red box and leaves the scene to the side again).*
	Control questions	• E1 (Control question 1): Where did the boy put the ball in the beginning?	• E1 (Control question 1): Where did the girl put her ball first?<br>• E1 (Control question 2): Where is the ball now?

		• E1 (Control question 2): Where is the ball now?<br>• E1 (Control question 3): Who put it there?<br>*Control questions 1-3 were repeated once when child answered incorrect, when child answered wrong again, E1 corrected her.*	• E1 (Control question 3): Who put it there?<br>*Control questions 1-3 were repeated once when child answered incorrect, when child answered wrong again, E1 corrected her.*
	Test question	E1: When the boy returns, where will he first look for his ball?	E1: When Tine returns, where will she first look for her ball?
Stimuli		Figurines (boy, girl), boxes	Silent, animated video with photographed figurines (boy, girl) and boxes
Accepted answer		Verbal answer or pointing	Verbal answer (when children pointed to the screen, they were asked to name the respective color of the box, if children were too shy to name the color, parents were asked where the child points to)

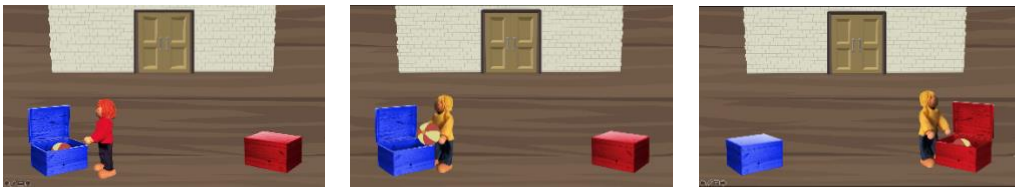

Figure 1. Sequence of screenshots of the silent video in Study 1 (False Belief Trial): (a) Girl puts ball in blue box. (b) Boy enters and takes ball out of the blue box in girl's absence. (c) Boy puts ball in red box in girl's absence.

Table 2

Detailed procedure of study 2

		Study 2	
		In Lab	Online
Script	Introduction	• E1: Look, [Name of the child], this is Smurf.<br>• E1 acts Smurf: Hello!<br>• E1: And this is his house. *(E1 puts little house made out of a paper box on the table).*<br>• E1: Look, Smurf and [Name of the child], what I have here. I have two boxes *(E1 shows two boxes).* Let's have a look inside the boxes *(E1 opens the two boxes).* This one is empty. And the other one is also empty *(E1 closes the two boxes again).* Okay, I put the two boxes here on the table.<br>• E1: And look, what I have here now. A toy car.<br>• E1 acts Smurf: Oh, great. A toy car! That is really great. I would love to have it. But I have to go into my house first, I have to do something.<br>• E1: Ok, then I put the toy car in here until then *(E1 puts toy car in one of the boxes).*<br>• E1 acts Smurf: Ahh, yes, okay. And now I have to go. *(Smurf goes into his house)*	• E1: Oh look, there is *Hedgehog.* Hello *Hedgehog! (Hedgehog comes into the scene)*<br>• Hedgehog *(voice recording in slide show)*: Hello!<br>• E1: Look, *Hedgehog* and [Name of the child], what I've got here. I have two boxes. *(Two boxes appear on the screen, one left and one right).* One box is purple and one box is orange. Let's have a look inside the boxes! *(Lid of the purple box lifts).* This box is empty. And this one? *(Lid of the orange box lifts).* It's also empty. And look, *Hedgehog* and [Name of the child], what I have here, a stone *(Stone appears on the screen).*<br>• Hedgehog *(voice recording in slide show)*: Oh great, a stone! That's great, I would love to have the stone. But wait, I have to go for a short moment.<br>• E1: Okay, I put the stone in here until then *(Stone moves in purple box).*<br>• Hedgehog *(voice recording in slide show)*: Aha, Ah ja! Ok,

		now I really have to go. *(Hedgehog leaves the scene)*
Location change	False Belief Trials:<br>• E1: Okay, [Name of the child]. Now we'll play a trick on *Smurf*. He can't see us now! Shhh…We put the toy car in this box now *(E1 takes the toy car out of the box and puts it in the other box).*<br>• E1: And now, we can call *Smurf* back. One, two, three… *Smurf*!<br>• E1 acts Smurf: *(Smurf comes out of his house into the scene again)*: Hello, I'm back!<br><br>True Belief Trials:<br>• E1: (waits first) Okay. Now, we call *Smurf* back again.. one, two, three, *Smurf*!<br>• Smurf: Hello, I'm back.<br>• E1: Watch carefully, *Smurf* and [Name of the child], what I'll do now. I take the toy car from the this box and put it in this box.<br>• E1 acts Smurf: Mh, yes, okay.	• E1: Okay, [Name of the child]. Now we'll play a trick on *Hedgehog*. He can't see us now! Shhh…We take the stone from the purple box and put it in the orange box now *(Human hand appears on the screen and puts the stone from the purple to the orange box).* Okay. And now, we call *Hedgehog* back again... one, two, three, *Hedgehog*!<br>• Hedgehog: *(Hedgehog comes into the scene, voice recording in slide show):* Hello, I'm back.<br>True Belief Trials:<br>• E1: (waits first) Okay. Now, we call *Hedgehog* back again.. one, two, three, *Hedgehog*!<br>• Smurf: Hello, I'm back.<br>• E1: Watch carefully, *Hedgehog* and [Name of the child], what I'll do now. I take the pencil from the green box and put it in the yellow box.<br>• Hedgehog *(voice recording in slide show)*: Mh, yes, okay.
Control questions	• E1 Control question 1 *(whispering to the child)*: Where did we put the toy car in the beginning?<br>• E1 Control question 2 *(whispering to the child)*: Where is the toy car now?	• E1 Control question 1 *(whispering to the child)*: In which box did I put the stone in the beginning?<br>• E1 Control question 2 *(whispering to the child)*: Where is the stone now?

		Control questions 1 and 2 were repeated once when child answered incorrect, when child answered wrong again, E1 corrected her.	*Control questions 1 and 2 were repeated once when child answered incorrect, when child answered wrong again, E1 corrected her.*
	Test question	E1 *(whispering to the child)*: Where does Smurf think that the toy car is?	E1 *(whispering to the child)*: Where does Hedgehog think that the stone is?
Stimuli		Figurine (Smurf, Sheep), boxes	Animated slide show with picture of protagonist (Hedgehog), voice recordings of protagonist and boxes
Accepted answer		Verbal answer or pointing	Verbal answer (when children pointed to the screen, they were asked to name the respective color of the box, if children were too shy to name the color, parents were asked where the child points to)

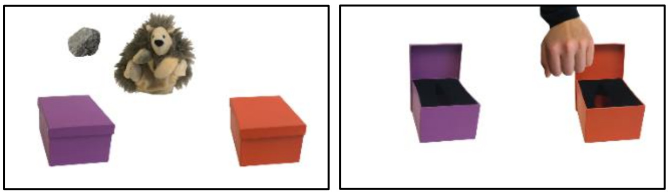

Figure 2. Screenshots of the animated slide show of Study 2 (False Belief Trial): (a) Stone is shown to child and *Hedgehog*. (b) Human hand moves stone from the purple to the orange box while *Hedgehog* is absent.

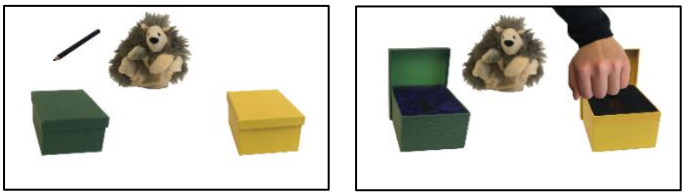

Figure 3. Screenshots of the animated slide show of Study 2 (True Belief Trial): (a) Pencil is shown to child and *Hedgehog*. (b) Human hand moves pencil from the purple to the orange box while *Hedgehog* is present.

References

Oktay-Gür, N., & Rakoczy, H. (2017). Children’s difficulty with true belief tasks: Competence deficit or performance problem? *Cognition*, *166*, 28–41. doi: 10.1016/j.cognition.2017.05.002

Schünemann, B., Schidelko, L. P., Proft, M., & Rakoczy, H. (2022). Children understand subjective (undesirable) desires before they understand subjective (false) beliefs. *Journal of Experimental Child Psychology*, *213*, 105268. doi: 10.1016/j.jecp.2021.105268
